# Emergency Detection in Smart Homes Using Inactivity Score for Handling Uncertain Sensor Data

**DOI:** 10.3390/s24206583

**Published:** 2024-10-12

**Authors:** Sebastian Wilhelm, Florian Wahl

**Affiliations:** Deggendorf Institute of Technology, 94469 Deggendorf, Germany; florian.wahl@th-deg.de

**Keywords:** emergency detection, ambient-assisted living, activity recognition, uncertain sensor data, inactivity score, smart home, IoT

## Abstract

In an aging society, the need for efficient emergency detection systems in smart homes is becoming increasingly important. For elderly people living alone, technical solutions for detecting emergencies are essential to receiving help quickly when needed. Numerous solutions already exist based on wearable or ambient sensors. However, existing methods for emergency detection typically assume that sensor data are error-free and contain no false positives, which cannot always be guaranteed in practice. Therefore, we present a novel method for detecting emergencies in private households that detects unusually long inactivity periods and can process erroneous or uncertain activity information. We introduce the *Inactivity Score*, which provides a probabilistic weighting of inactivity periods based on the reliability of sensor measurements. By analyzing historical Inactivity Scores, anomalies that potentially represent an emergency can be identified. The proposed method is compared with four related approaches on seven different datasets. Our method surpasses existing approaches when considering the number of false positives and the mean time to detect emergencies. It achieves an average detection time of approximately 05:23:28 h with only 0.09 false alarms per day under noise-free conditions. Moreover, unlike related approaches, the proposed method remains effective with noisy data.

## 1. Introduction

Globally, society is aging. In 1990, 6% of the world’s population was 65 years or older; by 2019, this figure had risen to 9%. Projections indicate that by 2050, approximately 16% of the global population will belong to this age group [1]. This demographic shift presents significant challenges, particularly in the care of the elderly [2].

As people age, their physiological functions deteriorate, making independent living increasingly difficult [2,3]. Nonetheless, elderly individuals often wish to live independently in their own homes for as long as possible, avoiding or delaying a move to a nursing home [4].

Numerous technological solutions have been developed to assist the elderly in maintaining their independence. These solutions are often grouped under the term Ambient-Assisted Living (AAL) and aim to support daily life or detect emergencies [5,6]. This paper focuses on an AAL system for emergency detection which identifies incidents of abnormal inactivity within the home, such as those caused by a fall. The system is specifically tailored to the needs of elderly individuals living alone, where the demand for automated emergency detection is especially relevant.

Studies indicate that approximately 30% of people over 65 fall at least once a year; for those over 80, this rate increases to around 50% [7,8,9,10]. Not every elderly person can help themselves up after a fall. Various authors reported that between 37% and 50% of the elderly who fall have difficulty getting up independently [7,11]. Therefore, it is necessary to call for assistance quickly after such an incident, especially when the elderly live alone [8,12].

In the literature, an event in which a person remains on the ground for more than an hour after a fall is called a ’long lie’ event. The frequency of such events is not definitively established and varies between 6% and 20% of fall incidents, depending on the target group and study methodology [13,14,15,16]. It is estimated that about 3% remain down for more than 6 hours and 1.5% for over 24 h [15,17].

Systems like emergency buttons, which individuals can wear to call for help, exist but are often not worn or used consistently [8,13,18,19,20]. Other solutions rely on wearable sensors such as smartwatches, ambient sensors such as fall mats, or radar sensors to detect falls actively. However, these often face user acceptance issues due to their intrusiveness and cost.

To address this issue, non-intrusive or minimally intrusive technology can be employed to analyze the behavior of the elderly, identifying deviations from typical behavior that might indicate an emergency. This technology leverages the fact that human daily routines typically follow a 24 h periodic rhythm—the circadian rhythm [21]. These rhythms follow quite consistent patterns for each household [22], and activity routines become increasingly entrenched with age [23].

Several existing studies consider deviations from typical behavior for emergency detection, often focusing on inactivity, i.e., unusually long periods without detected activity. We also apply this approach in our work. However, related works generally assume that the activity signals collected by sensors to create daily profiles are entirely reliable and trustworthy. With sensors that have certain errors in activity detection (e.g., due to pets triggering motion sensors) or those based on the disaggregation of existing data sources (which are also error-prone, as discussed in [24,25,26]), these algorithms reach their limits.

The main contribution of this work is introducing a novel approach to inactivity-based emergency detection using activity information that may contain uncertainties, with the primary target group being the elderly living alone. Furthermore, the newly proposed approach is benchmarked against four algorithms from the literature. It is shown that the approach presented in this paper outperforms related work in terms of the frequency of false-positive detection and mean time to emergency detection when both evaluation metrics are considered jointly. In particular, it is shown that the performance of the approach presented in this paper is only moderately affected by noise produced by uncertain sensors, which is unique, especially compared with related approaches.

The rest of this paper is structured as follows: Section 2 reviews related work on emergency detection based on activity information, focusing on inactivity detection algorithms. Section 3 presents a novel approach to emergency detection that can handle uncertain activity information by using an Inactivity Score (IS(t)). This approach is evaluated and benchmarked against four algorithms from related works in Section 4. Finally, Section 5 discusses the results, and Section 6 provides a summary and outlook.

## 2. Related Work

Detecting emergencies in private households is an intensively researched area with numerous approaches and commercial products [27,28,29,30]. These approaches mainly differ in the utilized sensors. Methods exist that rely on video or video-like signals [31,32,33,34,35], employ special pressure sensors such as fall mats [36], or use audio signals for emergency detection [37,38,39]. However, most approaches use sensors that detect binary activity events, such as Passive Infrared Motion Sensors (PIR) or door/window contacts [40].

This work also focuses on binary activity sensors. Algorithms based on these sensors for emergency detection can be divided into two categories: (1) algorithms to analyze residents’ behavior and detect anomalies [27,40] and (2) algorithms to analyze the absence of activities, i.e., inactivity [41]. The first category is particularly suited for detecting atypical behaviors, such as those due to dementia [21,40]. The second type of algorithm focuses on detecting (acute) emergencies, where a certain time interval between the emergency event and the alarm is always expected [42].

The main advantage of algorithms based on inactivity is that they only use binary information on whether an activity is present at a given time *t* [42]. Furthermore, the algorithmic complexity is lower compared with behavior-based algorithms. The foundation for these approaches is usually the Duration of Inactivity DI(t), as defined by Floeck and Litz [22]:

(1) DI(t):=elapsedtimeafterlastactivityevent

Cuddihy et al. [43] were among the first to address emergency detection by identifying unusually long periods of inactivity. They used a 30-minute rolling window to determine DI(t). Based on historical data, thresholds for each time interval were defined based on the highest usual DI(t), extended by a specific buffer. If this threshold is exceeded, an alarm is triggered. Weisenberg et al. [44] expanded this approach by integrating additional bed and chair sensors and portable actigraphs, increasing the system’s sensitivity.

Floeck and Litz [22,42] followed a similar approach but considered inactivity continuously. Alarm generation was based on a threshold determined by using a fourth-degree polynomial function. However, the work lacks a solid evaluation, which the authors have indicated as future work. We could not find any subsequent publications by the authors that include this evaluation.

In 2011, Floeck et al. [45] extended previous approaches and used a finite-state machine to monitor room stay duration instead of considering inactivity for the entire household. Stays of up to 20 min were considered unproblematic. If a person remained in a room for longer than 20 min, the probability of this was calculated based on historical data from the last 21 days. If this probability fell below 5%, the system triggered an alarm. The authors claimed that this approach could trigger an alarm 30 to 180 min after a potential emergency. However, the original work lacks a comprehensive evaluation, which the authors mention as a task for future work.

Moshtaghi et al. [46,47,48,49] similarly analyzed how long a person stays in a specific room or, more precisely, in a specific region of a house. It was checked at hourly intervals whether an abnormally long period of inactivity which exceeded a region- and time-dependent alert threshold was present. The alert threshold calculation was quite complex, involving analyzing historical inactivity data by using statistical models such as the Pareto and hyperexponential distributions. Adjustments and weightings based on historical data were implemented to ensure stable and reliable thresholds, along with continuous adaptation to changing behavioral patterns. Extensive post-processing steps were also included, such as filtering out unreachable thresholds.

In summary, existing approaches consider the time since the last activity for either the entire household or individual rooms and determine whether an emergency is present by using a threshold. They mainly differ in how the thresholds are generated. Further work, such as that by Valera et al. [50], focused on optimizing these thresholds.

None of the presented approaches are designed to handle uncertain activity signals. However, it is assumed that smart home-generated sensor measurements are inherently noisy, thus potentially providing erroneous information [27]. Another challenge, especially with motion detectors, are pets that can trigger activity [43]. If data from existing sources such as smart meters are disaggregated instead of using dedicated sensors to obtain activity information, false positives based on disaggregation are also to be expected [25,26,51].

This work aims to fill this research gap by presenting and evaluating a novel approach to detecting inactivity that can handle uncertain input signals.

## 3. Inactivity Score-Based Approach for Emergency Detection

As outlined in Section 2, several works exist that detect household emergencies based on inactivity. However, related work does not focus on handling uncertain activity signals. We introduce a novel approach to detecting emergencies based on activity signals that may contain some uncertainty. Unlike related works, we do not use DI(t), the Duration of Inactivity, also called the Inactivity Profile. Instead, we use a probabilistic approach and introduce the Inactivity Score IS(t). This approach assumes that the certainty $c\left(s_{j}\right)\in \left[0,1\right]$ for each sensor $s_{j}\in S$ is known, which can be derived from sensor specifications or a preliminary validation phase. However, determining these certainties accurately remains an open challenge in practice, as sensor performance may vary over time or due to environmental factors.

The following sections present the approach to creating an Inactivity Score IS(t), in Section 3.1, followed by the method for detecting anomalies—or emergencies—based on the Inactivity Score, in Section 3.2.

### 3.1. Inactivity Score

The Inactivity Score IS(t) is a metric that provides insights into the inactivity within a household at a specific time *t*. The Inactivity Score IS(t) is an adaptation of the Duration of Inactivity DI(t) introduced by Floeck and Litz [22], with the modification that activity events *e* do not reset the timer to zero unconditionally. This adaptation accounts for activity events detected by sensors that are not entirely reliable, i.e., those with $c(s_{j})<1$. This modification is particularly important in real-world scenarios, where sensor errors, false positives (e.g., caused by pets), or inconsistent data quality frequently occur. Practical systems must handle uncertain sensor readings due to various factors, such as temporary malfunctions, environmental conditions, or non-human movement within the home. While DI(t) tends to overreact to such uncertain signals, the Inactivity Score adapts to these variations, enabling the more accurate detection of genuine inactivity periods, particularly in environments with pets or inconsistent sensor performance.

It is important to note that IS(t) does not generally represent the inactivity duration at time *t*—analogously to DI(t) according to Floeck and Litz [22]. Instead, the Inactivity Score is a value ≥0, where a score of 0 at time *t* indicates absolute certainty of an activity at that time. A higher score signifies a longer period without confirmed activity or a greater probability of prolonged inactivity, considering uncertainties in the activity signals.

The score is calculated recursively for each time *t*, increasing by a slope defined as $a=\frac{\Delta t}{1\;\sec}$, similar to the definition in DI(t) by Floeck and Litz [22]. However, unlike DI(t), the reduction in the score at each time *t* is based on multiplication with a Reduction Factor $RF\left(t,E_{t}\right)\in \left[0,1\right]$. This Reduction Factor comprises all activity signals detected at that time *t*. Let $E_{t}$ be a set representing the sensor triggers in the interval [t−1:t]. The general formula for calculating the Inactivity Score IS(t) is given by Equation (2).

(2) $$IS\left(t\right)=\left(IS\left(t-1\right)+a\right)\times RF\left(t,E_{t}\right)$$

Unlike traditional DI(t), where any sensor event resets the inactivity duration to zero, the Inactivity Score reduces the current value by a factor to reflect the trustworthiness of each sensor at time *t*. Modeling trustworthiness enables the system to account for sensor uncertainties and improve robustness to noisy or unreliable data.

The Reduction Factor $RF(t,E_{t})$ for a given time *t* and a set of sensor triggers $E_{t}$ is calculated as shown in Equation (3). It is computed as the product of $\left(1-c\left(s_{j}\right)\right)$ for each sensor event and the individual Sensor Impact $I(t,s_{j})$ of the detecting sensor $s_{j}$ at time *t*. To capture simultaneous activity better, our score considers the combined impact of the activation of multiple sensors. If several independent sensors are triggered at the same time, the Inactivity Score is reduced more significantly.

(3) $$RF\left(t,E_{t}\right)=\prod_{s_{j}\in E_{t}}\left(1-c\left(s_{j}\right)\right)\times I\left(t,s_{j}\right)$$

The individual Sensor Impact $I(t,s_{j})$ depends on both the certainty $c(s_{j})$ of the sensor event and the time when the specific sensor $s_{j}$ was last activated before *t*, which is described as $l(t,s_{j})$. If the sensor was activated within a period $<\gamma (1-c\left(s_{j}\right))$, its influence is reduced. γ is a configurable hyperparameter which we set to γ=4 h in this work. If no sensor activation has occurred within γ, the Sensor Impact is set to 1. If activation has occurred within γ, a linear reduction by $\frac{t-l(t,s_{j})}{\gamma (1-c\left(s_{j}\right))}$ is applied. The reduced impact of sensors prone to frequent false triggers (e.g., due to malfunctions) prevents the system from being overly sensitive to unreliable events. The impact calculation is shown in Equation (4).

(4) $$I\left(t,s_{j}\right)=\left\{\begin{array}{cc} \frac{t-l(t,s_{j})}{\gamma (1-c\left(s_{j}\right))} & t-l\left(t,s_{j}\right)<\gamma \left(1-c\left(s_{j}\right)\right)\\ 1 & \mathrm{otherwise} \end{array}\right.$$

Thus, the overall formula for calculating the Inactivity Score IS(t) is summarized in Equation (5).

(5) $$IS\left(t\right)=\left(IS\left(t-1\right)+a\right)\times \prod_{s_{j}\in E_{t}}\left(1-c\left(s_{j}\right)\right)\times \left\{\begin{array}{cc} \frac{t-l(t,s_{j})}{\gamma (1-c\left(s_{j}\right))} & t-l\left(t,s_{j}\right)<\gamma \left(1-c\left(s_{j}\right)\right)\\ 1 & \mathrm{otherwise} \end{array}\right.$$

**Figure 1:**
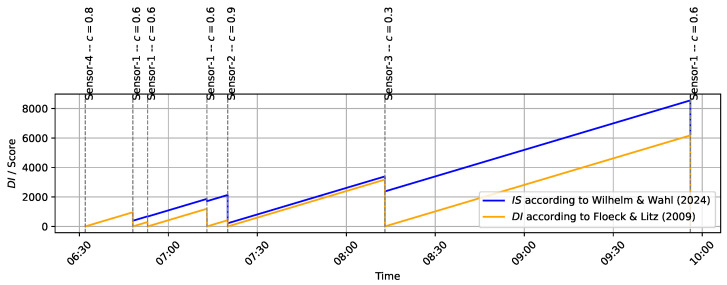
Example calculation of the Inactivity Score IS(t) according to Wilhelm and Wahl for the sample dataset in Table 1, compared with the Duration of Inactivity DI(t) according to Floeck and Litz [22].

Figure 1 demonstrates the calculation of IS(t) for the example dataset in Table 1. For direct comparison, the Duration of Inactivity DI(t) according to Floeck and Litz [22] is also shown. This example highlights the reduced influence of uncertain or frequently triggered sensors (e.g., Sensor-3 or Sensor-1 in quick succession at 06:48:00 and 06:53:00) on the Inactivity Score. In comparison, DI(t)) resets to zero with each event, regardless of the sensor’s certainty. This feature is especially important for emergency detection thresholds, as discussed in Section 3.2.

### 3.2. Emergency Detection Using the Inactivity Score

Emergency detection is based on historical scores. For each time *t*, a threshold thres(t) is calculated based on historical scores. An emergency is assumed if this threshold is exceeded. Thus, all times *t* are considered potential emergencies if

(6) IS(t)>thres(t)

The threshold calculation for time *t* is based on reference times in the past. A maximum reference period R(t) from t−ψ days to t−1 day, where ψ>1, is considered. Within this work, ψ is set to ψ=70 days. This reference window allows the system to dynamically adjust the threshold based on recent data, adapting to changes in the resident’s behavior.

The reference times $T_{ref}\left(t\right)$ are defined as

(7) $$\begin{array}{cc} T_{ref}\left(t\right)=\{t_{ref}\in R\left(t\right)| & \;\mathrm{time}\_\mathrm{of}\_\mathrm{day}\left(t_{ref}\right)=\mathrm{time}\_\mathrm{of}\_\mathrm{day}\left(t\right)\\ \wedge & \;\mathrm{is}\_\mathrm{weekend}\left(t_{ref}\right)=\mathrm{is}\_\mathrm{weekend}\left(t\right)\} \end{array}$$

For these reference times $T_{ref}\left(t\right)$, the maximum Inactivity Scores IS(t) within a window β before and after each reference time are calculated. The hyperparameter β is set to β=1 h in this work.

The maximum score $IS_{max}\left(t\right)$ is defined as shown in Equation (8).

(8) $$IS_{max}\left(t\right)=\left\{\max\left(IS\left(t_{k}\right)\right)|t_{k}\in \left[t_{\mathrm{ref}}-\beta ,t_{\mathrm{ref}}+\beta \right],\;t_{\mathrm{ref}}\in T_{\mathrm{ref}}\left(t\right)\right\}$$

To filter outliers in the threshold calculation, we define an upper bound Θ(t) based on the Interquartile Range (IQR) of the historical maxima $IS_{max}\left(t\right)$. The Θ(t) is calculated as shown in Equation (9).

(9) Θ(t)=Q3(t)+1.5×IQR(t)

where the following apply:

$$\begin{array}{cc} Q1(t) & =\mathrm{Percentile}(IS_{max}\left(t\right),25)\\ Q3(t) & =\mathrm{Percentile}(IS_{max}\left(t\right),75)\\ IQR(t) & =Q3(t)-Q1(t) \end{array}$$

The final threshold is based on the maximum of the $IS_{max}\left(t\right)$ values limited to Θ. A configurable scaling factor α is applied to allow for some variation beyond existing thresholds. We set the parameter for this paper to α=2.0. The threshold thres(t) is thus calculated as shown in Equation (10).

(10) $$thres\left(t\right)=\min\left(\max(IS_{max}\left(t\right)),\Theta \left(t\right)\right)\times \alpha$$

## 4. Evaluation

The evaluation of the proposed algorithm involved applying it to existing datasets containing activity information and benchmarking it against related algorithms. Specifically, benchmarks were conducted against the algorithms by Cuddihy et al. [43], Floeck and Litz [22,42], Floeck et al. [45], and Moshtaghi et al. [46], which were re-implemented. We ensured that the implementations closely followed the original papers. Hyperparameters were set according to the authors’ recommendations. The re-implementations and the implementation of the approach presented in this paper are available online at Code Repository: https://github.com/WilhelmSebastian/IBED (last accessed: 23 September 2024).

A core challenge of the evaluation is the lack of suitable datasets containing probabilistic activity signals from various sensor sources and sufficient emergency events. After extensive research, no such datasets were identified. To address this challenge, related datasets were modified to meet the requirements. We used the following approach: An activity dataset was utilized and modified to annotate the originally collected activity signals with a certainty value. Additionally, noise was created and added to the datasets.

The following datasets $D_{i}$ were modified accordingly:

- **CASAS, Aruba** https://casas.wsu.edu/datasets/aruba.zip (last accessed: 16 July 2024), and **CASAS, Aruba2** https://casas.wsu.edu/datasets/aruba2.zip (last accessed: 16 July 2024) [52]. These datasets contain sensor data collected in the home of a single resident. For the evaluation, only the ON-events of the motion sensors were considered. Due to strong correlations among sensors within the same room, only the motion sensors with the IDs `M007’, `M019’, `M020’, `M024’, and `M027’ were used. A rolling filter was applied, allowing only one sensor to be activated per hour to prevent frequent or nearly continuous trigger events by motion sensors when a person just stays in a room.
  Due to a data gap, only the sequence up to 2012-03-18 18:49:34 was used for *Aruba2*.
- **CASAS, Kyoto** https://casas.wsu.edu/datasets/kyoto.zip (last accessed: 16 July 2024) [53]. This dataset is similar to *Aruba* and *Aruba2* but was recorded in a different household with two residents. Here, similarly, only the ON-events of the motion sensors were considered, and a rolling filter was applied. The sensors used were `M007’, `M017’, `M020’, `M021’, `M029’, `M031’, `M038’, `M045’, and `M051’.
- **Wilhelm, Water HH-01, HH-05, HH-11, and HH-12** https://zenodo.org/records/7506076 (last accessed: 16 July 2024) [26]. These datasets contain water consumption data from various households measured by smart water meters. The data were analyzed and converted into activity data as presented by Wilhelm et al. [26]. Since all activity events were created by a single sensor, assigning activities to specific rooms was impossible. Due to larger measurement gaps, only the most extended sequence without a gap of more than one hour was considered for each dataset.

Table 2 lists the key properties of the individual datasets $D_{i}$.

From the individual datasets $D_{i}$, we obtained measurements from various sensors $S_{i}$. Each sensor $s_{i,j}\in S_{i}$ can be assigned to a specific room. However, all datasets $D_{i}$ lack the assignment function of individual sensors to a specific certainty value. Initially, it was assumed that $\forall s_{i,j}\in S_{i}:c\left(s_{i,j}\right)=1$, meaning that each sensor is fully trustworthy.

To simulate the performance of the proposed approach with erroneous data, various mapping functions $f_{n}^{D_{i}}:S_{i}\to \left[0,1\right]$, which assign different certainties to the individual sensors, were created. We considered four different noise levels n∈{N,L,M,H}, where the noise level determines how the certainties are distributed. The characteristics of the individual noise levels are described in Table 3.

The sampling process involved generating certainty values *c* from a normal distribution N(μ,σ) for each noise level. The values were then accepted only if they fell within predefined bounds specific to each noise level. This ensured that the sampled certainties realistically reflected the intended level of noise.

We denote the event set of dataset $D_{i}$ with the assigned certainties for noise level *n* as $A_{n}^{D_{i}}$. Additionally, a noise set $N_{n}^{D_{i}}$ was generated, containing $\left(1-c_{s_{i,j}}\right)\times \left|s_{i,j}\right|$ events for each sensor $s_{i,j}\in S_{i}$. The distribution of noise events over the day was based on the occurrence frequency of activation of the sensors in the original data $A_{n}^{D_{i}}$, supplemented by some global (time-independent) noise.

Based on the preprocessed evaluation data, two evaluation steps were performed: First, in Section 4.1, we analyze false-positive detection. Second, Section 4.2 examines the time taken to detect an emergency. These evaluation goals are fundamentally opposed, as typically, a lower false-positive rate results in increased detection time, while higher false-positive rates tend to lower detection time. Therefore, this chapter concludes with a comparison of the two evaluation steps in Section 4.3 to assess both metrics together in a balanced and comprehensive manner.

### 4.1. False-Positive Detection

The number of false-positive detection events was analyzed in the initial evaluation step. Reducing false positives is critical for emergency systems, as excessive false positives can lead to user dissatisfaction and potentially to the deactivation of the system [54].

Since the datasets $D_{i}$ used for evaluation do not contain any actual emergency events, every detected emergency was, by definition, classified as a false positive.

For the evaluation, the algorithms were applied throughout the entire duration of each dataset to detect potential emergencies. An initialization period of 10 weeks was used for each algorithm and dataset. False positives occurring during this period were not counted, as most algorithms, including the newly presented methodology, require a stabilization phase or are dependent on historical values (see, e.g., Moshtaghi et al. [46]).

Only the initial instance of a false positive was considered during the evaluation. Specifically, we focused on time points that satisfied the condition described in Equation (11). This criterion prevents overrepresentation caused by singular, prolonged false-positive events.

(11) $$\mathrm{FP}\left(t\right)=\left\{\begin{array}{cc} 1 & \mathrm{if}\;IS(t)>thres(t)\;\mathrm{and}\;IS(t-1)\leq thres(t-1)\\ 0 & \mathrm{otherwise} \end{array}\right.$$

**Figure 2:**
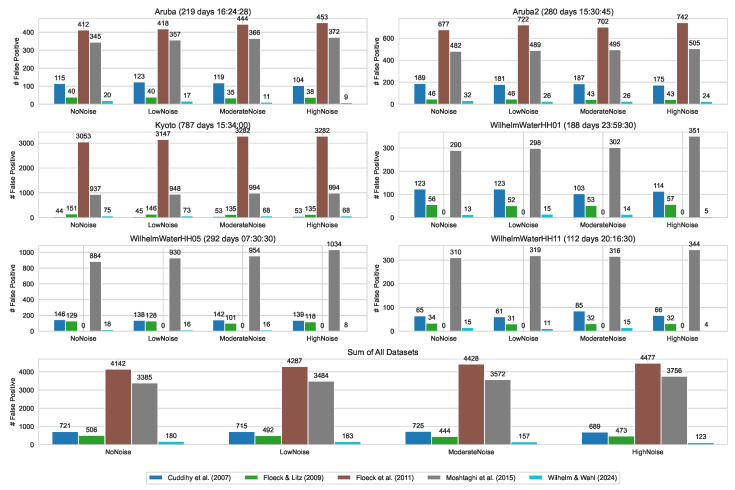
Number of false positives for each dataset–noise-level combination $A_{D_{i}}^{n}$ and for the sum of all datasets per algorithm. The absolute number of false positives after an initialization period of 10 weeks is shown [22,42,43,45,46].

Figure 2 shows the number of detected false-positive events after an initialization period of 10 weeks for each dataset–noise-level combination $A_{n}^{D_{i}}$ separately and summed across all datasets. Figure 2 shows significant differences between the individual algorithms and the various datasets. Across all datasets and regardless of the noise level, Floeck et al. [45] produced the most false positives, followed by Moshtaghi et al. [46], while the methodology presented in this work generated the fewest false positives.

Notably, Floeck et al. [45] produced false positives only in the *Aruba*, *Aruba2*, and *Kyoto* datasets, with no false positives in the *Wilhelm*, *Water* datasets. Similarly, Cuddihy et al. [43] produced fewer false positives than our proposed method for the *Kyoto* dataset. However, this result should be interpreted in the context of the second evaluation criterion, as fewer false positives often correspond to longer detection times or even missed detection events (see Section 4.2).

As shown in Figure 2, our method tended to produce fewer false positives as the noise level increased. This reduction is due to the algorithm dynamically adjusting its detection thresholds: as sensor errors become more frequent, the thresholds increase to maintain robustness against false alarms. Although this reduces the occurrence of false positives, it also leads to longer detection times, as discussed in Section 4.2.

### 4.2. Emergency Detection Time

The second evaluation step assessed the time to emergency detection. Minimizing this parameter is an essential quality attribute of the algorithm.

An emergency is assumed to occur when there is no more activity. Therefore, we simulated an emergency by stopping the activity signals from $A_{n}^{D_{i}}$ at emergency time $t_{e}$. The noise signals from $N_{n}^{D_{i}}$ were maintained to continue simulating errors. From $t_{e}$ onwards, the Inactivity Score IS(t) was calculated based solely on the inputs from $N_{n}^{D_{i}}$, and the time $t_{detect}$ was determined when the corresponding algorithm detected the simulated emergency event.

In the evaluation, we then considered the duration Δt from the emergency event $t_{e}$ to the detection of the emergency $t_{detect}$, where

(12) $$t_{detect}=\min\left\{t|t\geq t_{e}\wedge IS\left(t\right)>thres\left(t\right)\right\}$$

For each dataset–noise-level combination, we simulated 1000 emergencies randomly distributed over the entire dataset period (after an initialization phase of 10 weeks). We also limited Δt to a maximum of 7 days. If an algorithm was already in an alarm state at time $t_{e}$, this time point was excluded from the evaluation.

**Figure 3:**
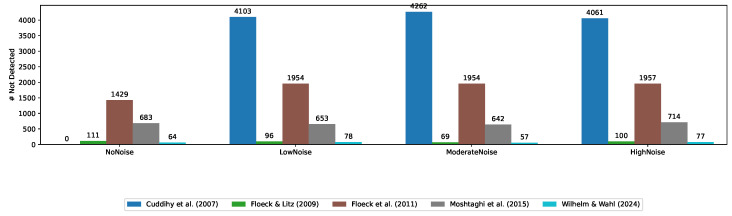
Number of undetected emergencies due to the restriction that events are excluded if Δt exceeds 7 days [22,42,43,45,46].

Figure 3 shows the number of emergencies excluded from the evaluation due to the restriction of a maximum Δt. It is particularly noticeable that many of the events were not detected by Cuddihy et al. [43], Floeck et al. [45], and Moshtaghi et al. [46].

**Figure 4:**
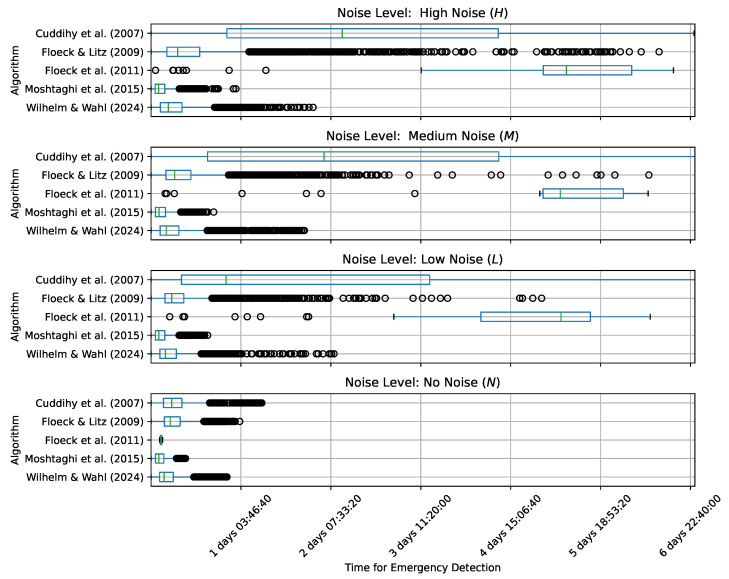
Boxplot showing Δt per noise level and algorithm for 1000 simulated emergency events across all evaluation datasets [22,42,43,45,46].

Table 4 shows the mean detection times per algorithm and noise level across all datasets $D_{i}$. Figure 4 further breaks down the results by noise level and algorithm in a boxplot.

Figure 3, Table 4, and Figure 4 must be interpreted together. Firstly, it is evident that the algorithms by Cuddihy et al. and Floeck et al. were significantly affected by the noise level, with low noise already causing detection times of over 2 and 4 days, respectively. Similarly, the number of false negatives increased significantly for these algorithms as the noise level increased.

In contrast, the algorithm by Floeck and Litz was notably less susceptible to noise, with fewer non-detection events. However, Figure 4 shows that with the increase in the noise level, there were numerous outliers, with detection time extending over several days.

The approach presented in this work is characterized by the fact that the noise level only rarely resulted in non-detection events while the mean detection time still increased at a moderate rate. Nevertheless, there were a few outliers, with detection time exceeding one day. Moshtaghi et al. had the shortest detection time. Their algorithm seemed barely affected by noise levels in terms of detection time but had numerous non-detection events, across all noise levels.

### 4.3. False Positives vs. Emergency Detection Times

In the previous subsections, false-positive detection and emergency detection times were considered independently. However, it is expected by definition that these two evaluation goals are inversely related, that is, a lower false-positive detection rate usually negatively impacts the detection time, and vice versa. In most algorithms, including the one presented in this paper, a hyperparameter that determines whether the algorithm is expected to have shorter detection time or generate fewer false positives can be set.

**Figure 5:**
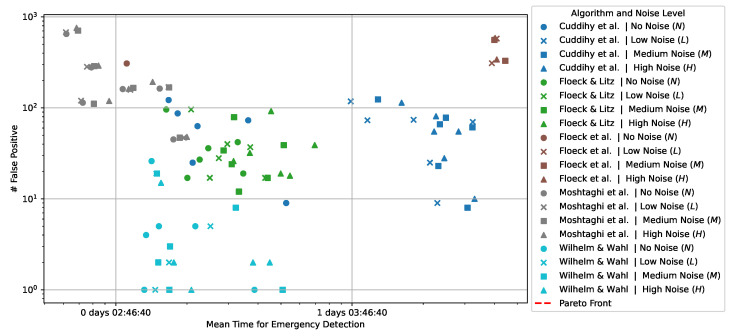
Comparison of emergency detection algorithms by noise level: number of false positives vs. mean detection time on a logarithmic scale [22,42,43,45,46].

Figure 5 plots the number of false positives and the detection time for the different algorithms across all evaluated datasets. The presented methodology is represented by the light-blue cluster at the bottom on the left, indicating that it outperformed the related works across all noise levels when both evaluation goals were considered. The gray cluster at the top left represents the work of Moshtaghi et al. [46], showing the shortest detection times across all levels but also the highest number of false-positive classifications. Floeck et al. [45] showed the poorest performance.

In conclusion, our method consistently strikes a balance between minimizing false positives and maintaining reasonable detection time, even under high-noise conditions.

## 5. Discussion

In our work, we developed a novel methodology to detect emergencies in households based on residents’ inactivity, even when the input data contain uncertain or incorrect activity signals. This feature is crucial for real-world applications, as sensor data in real environments can be partially incorrect due to various factors, such as pets, malfunctions, or the reliability of the data source itself [25,27,43].

In contrast to related approaches by Cuddihy et al. [43], Floeck and Litz [22,42], Floeck et al. [45], and Moshtaghi et al. [46], the methodology presented in this paper does not rely on the Duration of Inactivity (DI(t). Instead, it employs a newly developed Inactivity Score (IS(t)), described in Section 3.1. This score makes the algorithm less susceptible to faulty or uncertain data.

**Figure 6:**
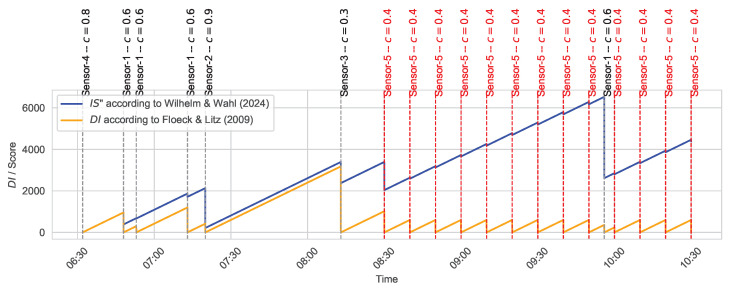
Comparison of Inactivity Score (IS) and Duration of Inactivity (DI) in the presence of a faulty sensor.

The Inactivity Score (IS(t)) is also designed to handle cases where sensors may have malfunctions and continuously provide incorrect activity signals. Figure 6 illustrates how the Inactivity Score (IS(t)) compares to the classical Duration of Inactivity (DI(t)) according to Floeck and Litz [22,42], particularly when a faulty sensor (e.g., Sensor-5) frequently triggers (faulty) activity signals. The Inactivity Score (IS(t)) significantly limits the influence of the faulty sensor, whereas the Duration of Inactivity (DI(t)) is prevented from increasing, ultimately hindering emergency detection.

However, the assumption that sensor certainty values can be accurately determined and assigned presents an open challenge in practice. Sensor certainty may fluctuate over time due to environmental factors, hardware degradation, or other external influences.

In addition to using the Inactivity Score as a basis, our approach differs from related work in how thresholds are calculated. While most related works base their thresholds on historical values, these are typically limited to the ’hour of the day’ [22,42,43,44] or, in some cases, include room- and time-dependent thresholds [45,46]. None of these approaches consider the day of the week. Our approach incorporates this feature into the threshold calculation, assuming that daily rhythms vary depending on the day of the week, particularly between weekends and weekdays. This assumption is also supported by the literature [31,42]. Moreover, since the threshold is based on a sliding window of the past ψ days, the system can adapt to changes in behavior over time, such as seasonal variations. While our approach does not consider room-specific activity, as performed by Floeck et al. [45] and Moshtaghi et al. [46], this could be an avenue for future enhancements.

One of the key characteristics of the proposed approach is its recursive nature. The Inactivity Score at time *t* is always built upon the Inactivity Score from t−1. The straightforward structure—involving a multiplication of several factors—enhances the explainability of the model, which is an important aspect for decision-making systems, especially in healthcare applications. However, due to the recursive design, a continuous computation of the current Inactivity Score is necessary. However, the calculation for each time step requires only minimal computational resources. This reduced complexity also facilitates deployment on resource-constrained edge devices, enabling real-time computations directly within households while maintaining privacy by avoiding cloud-based processing.

The evaluation of our approach (see Section 4) demonstrates that we have successfully developed an emergency detection algorithm capable of operating with binary activity signals and tolerant of sensor uncertainties and faulty data. When considering both evaluation dimensions, i.e., the number of false-positive detections and the mean emergency detection time, the algorithm presented in this paper outperforms related works in the absence and presence of noise.

It is also important to note that aside from Moshtaghi et al. [46], none of the authors of the examined approaches extensively evaluated their methods in their original papers. Even Moshtaghi et al. performed worse in our benchmark than their original evaluation, likely due to the smaller dataset used in our evaluation.

However, it must be mentioned that a mean detection time of over 7 h for detecting emergencies is long when individuals need acute assistance. Therefore, the presented methodology should not be considered a primary emergency detection system but rather a supplementary one. It serves as a fallback system capable of detecting emergencies when other AAL systems, such as wearables or emergency buttons, fail due to misuse, dead batteries, or similar issues. This reduces the time someone might remain unattended after an emergency.

The system is particularly suited for individuals living alone, offering a cost-effective solution by utilizing existing but potentially uncertain data sources, such as smart meters. However, it should be noted that more advanced technologies—those that go beyond inactivity detection and provide comprehensive behavioral analysis—could detect a wider range of emergencies or even predict early signs of illness or other critical conditions.

It is critical to note that the algorithm presented here depends on numerous hyperparameters (especially γ,ψ,β, and α). The certainty for each sensor must also be known or determined by experts. In this work, we used expert knowledge to establish standard parameter settings across all datasets and households. However, these parameters should be optimized for each specific dataset or household.

One of the main limitations of this work is that the evaluation was conducted by using simulated noise data and assumed sensor certainties due to a lack of real-world datasets. Future work should focus on testing the approach in practical environments with accurately collected, non-simulated activity data. Further investigation is needed to determine how the necessary hyperparameters and sensor certainties can be optimized automatically. A self-learning system based on an initialization phase of several weeks would be conceivable.

## 6. Conclusions and Outlook

In this work, we presented a novel approach for detecting emergencies in private households based on the Inactivity Score (IS(t)). Unlike the Duration of Inactivity (DI(t)) commonly used in related work, the Inactivity Score weights individual activity events, allowing for the use of activity signals from sensors with uncertainties.

The developed approach for emergency detection delivered convincing evaluation results. In evaluations with seven different datasets, the algorithm could detect simulated emergencies with entirely certain sensor signals with a mean detection time of 05:23:28 h, producing 0.09 false positives per day (without noise). Based on simulated noise data, which represent uncertain sensors, it was demonstrated that the performance of the proposed approach is only moderately affected by noise, which is unique compared with related approaches. Even with a high noise level and an average sensor certainty of 0.85, the algorithm detects an emergency with a median time of 07:16:23 h and produces 0.06 false positives per week.

Overall, our novel methodology significantly advances the state of the art in emergency detection in private households and demonstrably brings substantial quality improvements. The approach’s tolerance to faulty activity signals makes it more practical than previous methods and addresses existing limitations. In particular, fall or ’long lie’ events can be detected well with the presented algorithm, potentially mitigating health risks.

Future work should focus on deploying and evaluating our proposed approach in practical environments, particularly with accurately collected, real-world activity data. Further investigation is also necessary to explore the automatic optimization of hyperparameters and learning of sensor certantiy values. One promising direction could involve using feedback from false-positive alarms to refine sensor certainty estimates, allowing the system to dynamically adapt to changing sensor reliability. A self-learning system based on an initialization phase of several weeks would be conceivable.

## Figures and Tables

**Table 1:** Example dataset with 4 sensors for demonstration purposes.

Time	Sensor	Certainty
06:32:00	Sensor-4	0.8
06:48:00	Sensor-1	0.6
06:53:00	Sensor-1	0.6
07:13:00	Sensor-1	0.6
07:20:00	Sensor-2	0.9
08:13:00	Sensor-3	0.3
09:56:00	Sensor-1	0.6

**Table 2:** An overview of the key properties of the datasets $D_{i}$ used for evaluation.

Dataset	#Sensors	#Events	Days Covered
CASAS Aruba	5	2861	≈220 days
CASAS Aruba2	5	4146	≈281 days
CASAS Kyoto	9	13,501	≈788 days
Wilhelm, Water HH-01	1	3731	≈189 days
Wilhelm, Water HH-05	1	13,773	≈292 days
Wilhelm, Water HH-11	1	3843	≈113 days
Wilhelm, Water HH-12	1	1099	≈111 days

**Table 3:** Noise levels and their descriptions.

Noise Level	Description
No noise (*N*)	c∼N(μ=1.00,σ=0.00);1.0≤c≤1.0
Low noise (*L*)	c∼N(μ=0.95,σ=0.02);0.9≤c≤1.0
Medium noise (*M*)	c∼N(μ=0.90,σ=0.10);0.8≤c≤1.0
High noise (*H*)	c∼N(μ=0.85,σ=0.20);0.6≤c≤1.0

**Table 4:** Mean time of Δt per noise level and algorithm for 1000 simulated emergency events across all evaluation datasets.

Algorithm	No Noise (*N*)	Low Noise (*L*)	Medium Noise (*M*)	High Noise (*H*)
Cuddihy et al. [43]	0 days 07:44:37	2 days 01:09:09	2 days 17:00:13	2 days 19:36:40
Floeck and Litz [22,42]	0 days 06:59:19	0 days 08:26:53	0 days 10:09:28	0 days 13:17:45
Floeck et al. [45]	0 days 03:05:23	4 days 15:00:54	4 days 22:27:07	4 days 16:54:23
Moshtaghi et al. [46]	0 days 02:55:34	0 days 03:05:59	0 days 03:11:37	0 days 03:10:15
Wilhelm and Wahl	0 days 05:23:28	0 days 06:09:02	0 days 06:48:56	0 days 07:16:23

## Data Availability

The datasets utilized in this study are publicly available. The CASAS Aruba dataset can be accessed at https://casas.wsu.edu/datasets/aruba.zip, the CASAS Aruba2 dataset at https://casas.wsu.edu/datasets/aruba2.zip, and the CASAS Kyoto dataset at https://casas.wsu.edu/datasets/kyoto.zip. Additionally, the Wilhelm, Water dataset is available at https://zenodo.org/records/7506076. All datasets were last accessed on 16 July 2024. The source code for the re-implementations of the related approaches and the implementation of the approach presented in this paper is available at https://github.com/WilhelmSebastian/IBED (last accessed: 23 September 2024).

## Abbreviations

The following abbreviations are used in this manuscript:

AAL	Ambient-Assisted Living
PIR	Passive Infrared Motion Sensors
IQR	Interquartile Range
